# Cognitive Improvement in Older Adults with Mild Cognitive Impairment: Evidence from a Multi-Strategic Metamemory Training

**DOI:** 10.3390/jcm9020362

**Published:** 2020-01-28

**Authors:** Jung-Hae Youn, Soowon Park, Jun-Young Lee, Seong-Jin Cho, Jeongsim Kim, Seung-Ho Ryu

**Affiliations:** 1 Department of Counseling Psychology, Cha University, Gyeonggi-do 11160, Korea; jung_et@naver.com; 2 Department of Education, Sejong University, Seoul 05006, Korea; spark@sejong.ac.kr; 3 Department of Psychiatry and Neuroscience Research Institute, Seoul National University College of Medicine & SMG-SNU Boramae Medical Center, Seoul 07061, Korea; kimjeongsim83@gmail.com; 4 Department of Psychiatry, Gil Medical Center, Gachon University College of Medicine, Incheon 21565, Korea; sjcho@gilhospital.com; 5 Department of Psychiatry, School of Medicine, Konkuk University, Konkuk University Medical Center, Seoul 05030, Korea; shryu@kuh.ac.kr

**Keywords:** multi-strategic metamemory training, mild cognitive impairment, random control design, transfer effect

## Abstract

Intervention programs to relieve memory impairment and memory-related complaints in older adults with mild cognitive impairment are needed. Objective: The purpose of the current study was to assess the efficacy of a novel cognitive training approach—named multi-strategic metamemory training—in older adults with amnestic mild cognitive impairment. Among a total of 113 older adults with mild cognitive impairment, 66 participated in the memory training program (training group) and 47 did not (control group). Repeated measures of analysis of variance revealed that compared with the control group, the training group experienced: (i) a significantly greater increase in cognitive test scores of long-term delayed free recall (*F*_interaction_ = 6.04, *p* = 0.016) and fluency (*F*_interaction_ = 4.11, *p* = 0.045) and (ii) significantly greater decrease in their subjective memory complaints for everyday memory (*F*_interaction_ = 7.35, *p* = 0.009). These results suggest that the training program can improve verbal memory (i.e., delayed free recall), language processing (i.e., categorical fluency) and limit complaints in everyday instrumental memory activities of mildly impaired older adults.

## 1. Introduction

The proportion of older adults is growing rapidly, making it increasingly important to understand and intervene in patients with cognitive decline accompanied with normal or pathologic aging. Researchers have demonstrated that cognitive impairment may be postponed or minimized through cognitive training programs; these programs may be effective in normal older adults and importantly, those with amnestic mild cognitive impairment (aMCI). Paying close attention to older adults with aMCI is critical because: (i) aMCI is considered a potential prodrome for Alzheimer’s disease [1] and (ii) mildly impaired cognitive function could increase subjective complaints in everyday life. Therefore, there have been a number of programs designed to develop effective interventional programs for older adults with aMCI to prevent further cognitive decline. 

Memory notebook is one of the classical trainings to aid functional independence in everyday life in patients with memory deficits [2]. It gives external assistance to compensate specific problems in real life (e.g., scheduling appointment) and support to increase functional independency in amnestic patients, but the objective memory score (i.e., recall) was not changed by the Memory notebook training [3]. Training programs leveraging an understanding of memory and self-awareness of memory processing of participants, accompanied with multi-mnemonic strategies (e.g., story making, imagination, associations) may help enhance cognitive function in older adults [4]. Factual knowledge about memory and aging is likely to be a great strategy for enhancing memory function in older adults. For instance, the Memory and Aging Program (MAP) educate global knowledge about memory and aging, memory strategies such as spaced retrieval and semantic association [5]. After the 5-week training, knowledge about aging, number of memory strategies, satisfaction with one’s memory ability and self-reported everyday memory functioning were increased in the training group than the control group, whereas the objective memory performances (i.e., recall of word-list, recall of names) showed no difference between the groups. The MAP focused on the memory strategies in everyday life (e.g., locations of items, remembering numbers), but training focused on the superordinate memory strategies (e.g., story-telling, visualization, organization, method of loci through) as well as strategies in everyday life is needed to find effects on objective memory performance.

The multi-strategic metamemory training (MMT) encompassed knowledge of one’s own memory processes and superordinate memory strategies [4]. Healthy older adults who participated MMT improved more substantially compared with the wait list control group in trained areas and non-trained areas [4]. A follow up study with a larger sample size of healthy adults and brain imaging replicated the transfer effects of the MMT and brain structural changes further support the initial evidence of these effects [6]. These previous results suggest that metamemory, an understanding of how memory works and monitoring of one’s memory processing, could facilitate the effects of mnemonic strategies and lead to improvement of older adults’ cognitive function including memory function [7,8]. Therefore, it can be hypothesized that MMT would lead to improvement of cognitive function in older adults with MCI by increasing meta-cognitive function. Furthermore, we also anticipated that the subjective memory complaint in everyday life can be reduced, because the MMT covered memory training in practical circumstances in every 10 sessions. Based on the previous study that subjective memory complaint was not related to the objective memory performance in older adults with MCI [9], we thus assumed that memory training in practical circumstances can have more positive effects on the subjective memory complaint rather than the negative effects of awareness of their memory deficit on the subjective memory complaint. Randomized and controlled studies with large sample sizes of individuals with MCI are needed to quantify and characterize intervention effects. After a systematic review of 15 cognitive interventions for individuals with MCI, Jean et al. noted that: i) 14 out of these 15 previous studies recruited fewer than 30 participants in the training group and ii) only five out of these 15 studies adapted a robust research design, namely, a randomized control design, to characterize the effect of the training [10]. Although developments relating to cognitive improvement in older adults with MCI are consistently reported in the literature [11], the extent of the effects and the ability to generalize results are still not well elucidated. 

The current study aimed to assess the effect of MMT in older Korean adults with MCI. We utilized a randomized control design study with 113 older adults with aMCI (i.e., 66 older adults in the training group, 47 in the wait list control group). We hypothesized that the MMT would lead to improvements of cognitive functions in older adults with aMCI and also had a positive effect on their subjective memory complaints. 

## 2. Methods and Measures

### 2.1. Participants

A total of 154 older Korean adults (age range = 60–85 years) with aMCI were recruited from a Seoul National University Boramae Medical Center and a community center, but 12 were dropped in the training group and 29 were dropped in the control group, because they fell out of contact with the researchers. The remaining 113 (66 in the training group, 47 in the control group) participated in the current study. aMCI diagnostic criteria were defined by Petersen et al. in 1997 [12]; (1) a memory complaint, preferably confirmed by an informant; (2) memory impairment, below one standard deviation than those of age- and education-matched healthy individuals by Consortium to Establish a Registry for Alzheimer’s Disease Neuropsychologischen Testbatterie (CERAD-NP); (3) normal general cognitive function; (4) overall intact activities of daily living; (5) without dementia; (6) without other evident medical, neurological or psychiatric explanations for the memory impairment. 

Demographic and neuropsychological characteristics of participants are presented in Table 1. Exclusion criteria included: (i) known history of brain tumor, stroke, head trauma, cerebral infection or any illness that could affect neurocognitive function, (ii) visual, hearing or speech and language impairment that could cause problems following directions, (iii) known history of alcohol or drug abuse, or (iv) brain imaging that indicated any possibility of brain lesions other than MCI. 

### 2.2. Multi-Strategic Metamemory Training (MMT)

The MMT approach is based on the metamemory concept and was developed by Youn et al. [4]. Metamemory is a type of metacognition and it involves knowledge of one’s own memory strategies, introspective process and the capabilities of monitoring and controlling one’s own memory process. MMT was basically designed to develop metamemory function. In the MMT, participants study how to cope with forgetting and learn how memory strategies operate. Participants also have time to monitor their own memory process. 

The trained researchers administered the training using pen and paper. An average 80% of participants finished the MMT. The training consists of 10 sessions with 1-week intervals; each session takes 90 min. Each session is divided in three parts, which take 30 min each. The first part addresses the storage of knowledge as memory according to the metamemory model; the second part addresses training of memory methods based on metamemory; the third part covers memory training in practical circumstances. Metamemory is a subcategory of metacognition comprised of one’s understanding of their memory strategies and the awareness and regulation of their memory. MMT allows someone to understand how memory strategies work within the mind, handle memory loss with age and better implement memory strategies in everyday circumstances. MMT further comprises meta-monitoring, enabling one to track their personal quality of memory, implement memory strategies in their everyday lives and compare experiences with one another in each session.

The program is introduced in the initial session and participants compare their experiences of memory loss. The instructor provides an overview of MMT and teaches the participants how to better use memory strategies and cognitive training in their everyday lives. Finally, to introduce themselves, program participants discuss their experience with memory loss, when they had been inconvenienced by degradation in their ability to remember and how they had handled each incident, recognizing the commonality of inconvenience, embarrassment, fear of old age, depression, anxiety and family problems associated with memory loss.

The instructor explains the dynamics of energy loss in the second and third sessions, inspiring participants to immerse themselves in the training by pointing out that memory loss accelerates with age because as the working memory span declines incrementally over a protracted period, information encoding and retrieval proceeds at a constant rate as we age. Next, the instructor teaches the participants to handle memory loss by using metamemory. Training in a metamemory-based strategy enables the participants to acquire skills to encode information with their narrower working memory span and retrieve memory slower, improving participants’ memory strategies. Furthermore, participants can observe their present states through meta-monitoring with their improved meta-cognition and discuss them with each other. Finally, the participants are assigned to implement the strategies for dealing with memory loss in their everyday circumstances. The second session’s coping method and training exercise is for each participant to name their current activity out loud; the third session requires that participants conjure a mental image of their activities.

The instructor begins Part 1 of Sessions four through nine with explanations of the structure of memory (Sessions four and five); attention (Session six); the structure and function of the brain (Session seven); the environment (Session eight); perception (Session nine). Participants are instructed in memory strategy processes in Part 2. The instructor educates the participants on memory strategies such as story-telling, visualization, organization and method of loci through a number of visual media and other educational materials. Participants discuss the real-life application of the memory strategies acquired in prior sessions in Part 3, and are given assignments through which to apply the strategies.

The 10th session is a conclusion in which highlights of prior sessions are presented, and participants give feedback on the training and experiences of memory and its failure. During this exchange of ideas, the session administrator urges participants to consistently practice and utilize the memory strategies in their everyday circumstances. More information has been described in previous studies [4,13].

### 2.3. Mini Mental State Examination (MMSE) 

The MMSE was a test designed to assess cognitive functions [14]. The Korean version of the MMSE was developed and validated in older Korean adults by Lee et al. [15]. The orientation (10 points), short-term memory registration and recall (6 points), attention (5 points), naming (2 points), following verbal commands (4 points), judgment (2 points) and copying a double pentagon (1 point) were assessed. The score ranged from 0 to 30 and higher scores indicated better cognition. Generally, Korean older adults with aMCI showed average score of 26.51 [16].

### 2.4. Elderly Memory Disorder Scale (EMS) 

The EMS was developed and validated to measure memory and cognitive functions in older Korean adults [17]. In the EMS, Elderly Verbal Learning Test (EVLT) the Simple Rey Figure Test (SRFT), the Boston Naming Test (BNT), Categorical Fluency Test (CFT), Digit Span test (DST) and Spatial Span test (SST) were included. In the EVLT, nine words from three categories were freely and cued recalled immediately and after a 20-min delay. Scores ranged from 0 to 9 per condition (i.e., immediate free recall, immediate cued recall, delayed free recall, delayed cued recall). In the SRFT, 16 figures were immediately copied, and freely recalled and recognized after a 20-min delay. Scores ranged from 0 to 16 for copying and freely recall; 0 to 20 for recognition. In the BNT, participants were asked to name 15 pictures, thus, the score ranged from 0 to 15. In the CFT, participants were asked to name as many animals, fruits, colors and cities as possible in one minute each. In the DST, number digits were recalled in forward and backward. In the SST, tapped sequences at the identical blocks were recalled in forward and backward. 

### 2.5. The Subjective Memory Complaints Questionnaire (SMCQ) 

The Subjective Memory Complaints Questionnaire (SMCQ) was developed and validated in the Korean population [18] to evaluate subjective memory complaints. The SMCQ included 14 items; four assessed the global memory function and 10 assessed everyday memory function. Global memory function measured subjective complaint about metacognition of general, whereas everyday memory measured subjective complaint about specific memory function in everyday life. Examples for the global memory function were “Do you think that you have a memory problem?” and “Do you think that your memory is poorer than that of other people of a similar age?”. Examples for the everyday memory function were “Do you have difficulty in remembering two or three items to buy when shopping?” and “Do you have difficulty in remembering an appointment made a few days ago?”. Participants answered either yes (1) or no (0). Higher scores indicated a greater perceived memory complaint. 

### 2.6. Procedures 

The participants were recruited from February 2011 to February 2013. Participants who agreed to participate in the study and were diagnosed as having aMCI were randomly assigned to the training group and the control group. The randomization assigning process was carried out by generating random number using Excel. If the generated digit was odd, the participant was assigned to the training group; if the generated digit was even, the participants was assigned to the control group. The control group of the current study was on the waiting list, and had no lectures or activities. Neuropsychological measures (i.e., MMSE, EMS) were conducted twice, prior to the training (pre-training evaluation) and after the training (post-training evaluation). The interval between the pre-training and the post-training was 9 weeks. Clinical researchers who were blind to the group of participants assessed neuropsychological measures. All participants provided written consent before participating in the study and the study was conducted in agreement with the Declaration of Helsinki and approved by the ethics review board of Seoul National University Boramae Medical Center. Participants received no rewards or payments.

### 2.7. Statistical Analysis

A SPSS 18.0 for Windows (Chicago, IL, USA) was used for data analyses. Differences in demographics and neuropsychological assessment (i.e., age, education, gender, MMSE) between groups were tested using independent *t*-tests or *χ*^2^ test. Repeated-measures of analyses of variance (rmANOVA) was conducted to assess differences in changes of neuropsychological scores (i.e., EMS, SMCQ) between pre and post training across the training and control groups. Statistical tests were two-tailed, with *p* < 0.05.

## 3. Results

### 3.1. Homogeneities in Demographics and Psychiatric Characteristics across Groups

Demographic and psychiatric characteristics of participants in the training group and the control group are presented in Table 1. There were no differences between the groups in gender distribution, age, education, depressive symptoms and general cognitive functions measured by MMSE. Furthermore, independent *t*-test revealed that there were no differences in any measure of cognitive functions in the EMS prior to training across the groups (all *p*s > 0.05). 

### 3.2. Changes in Cognitive Functions Across Groups 

Table 2 presents descriptive statistics and notes differences among the groups in changes in cognitive functions. Among the scores, a significant interaction between the score change and groups (i.e., training group, control group) were found for tests of verbal memory (i.e., delayed free recall; *F*_interaction_ = 6.04, *p* = 0.016), language (i.e., fluency; *F*_interaction_ = 4.11, *p* = 0.045) and subjective memory complaint (i.e., everyday memory; *F*_interaction_ = 7.35, *p* = 0.009). The delayed free recall and fluency scores increased significantly more in the training group compared with the control group, whereas the everyday memory complaint was significantly decreased in the training group compared with the control group. The interactions in the scores of other measures (i.e., Immediate free recall, Immediate cued recall and delayed cued recall of EVLT; copy, recall and recognition of SRFT; BNT; forward, backward score of DST; forward, backward score of SST; total and global score of SMCQ) were not significant, which indicate that the changes across groups (i.e., training group, control group) were not significantly different (all *p*s > 0.05).

## 4. Discussion

The current study demonstrates that older Korean adults with MCI experienced improved memory (i.e., delayed free recall), language function (i.e., categorical fluency) and reductions in their everyday memory complaint after participating in ten weeks of MMT. The current study broadens the understanding of memory intervention in older adults with MCI by providing evidence that the objective memory performance could be enhanced by the educational approach about the memory and teaching memory strategies. 

Visuospatial memory and attention were not significantly different between the training and control groups. The results of the current study are similar to a previous study that noted the effects of MMT in normal older adults [6]. Youn et al. found that older adults who participated in the MMT (*n* = 112) experienced significantly greater increases in delayed free recall and language functions (i.e., fluency, BNT) compared with the control group (*n* = 89). Of note, the previous study also found no differences in visuospatial memory and attention between the training and control groups. Taken together, these results suggest that the MMT may lead to improvements in verbal memory rather than visuo-spatial memory or attention. 

Verbal memory function is related with the left medial temporal lobe, while the function of non-verbal memory (e.g., visuo-spatial memory) is related with the right medial temporal lobe [19]. A previous study found that MMT-induced changes in the brain were more significant in the left hemisphere compared with the right [6]. For instance, they found that mean diffusivity of left superior longitudinal fasciculus, left external capsule or left corona radiata were more significantly decreased in the training group compared with the control group. As the brain-imaging data from the previous study and behavioral data from the current study suggests, MMT may effectively improve verbal memory function in both normal and mildly cognitive impaired older adults. 

Among the verbal memory function, improvements were significant in the delayed free recall, but not the short-term free recall, short-term cued recall or delayed cued recall. This result suggests that the MMT helps older adults with MCI to store memories more efficiently and improve the retrieval process in a situation with no cues. This may be possible because the MMT dealt with mnemonic skills, an approach which can help improve memory consolidation and lead to participants reviewing their memory processes. 

Categorical fluency was improved after participating in the MMT. The language function (i.e., categorical fluency) was not directly covered in the MMT. The BNT also showed improvement only in the training group, even though the difference was not significant. These results revealed that the MMT has a transfer effect on language processing. Researchers demonstrated that the training effects are limited to the trained cognitive function (e.g., memory) and the evidence for the transfer effect is minimal [20]. However, categorical fluency was not directly trained in MMT, thus, the MMT is more effective than other trainings that are not having any transfer effect. One possible explanation about this transfer effects may be the categorical fluency requires efficient retrieval processes and semantic memory [21,22]. The MMT focused on the memory processes and the results showed that the delayed free recall, which reflects efficiency of retrieval processes, was improved by MMT. There may be shared mechanisms that played a role to enhance in delayed free recall and the categorical fluency. This explanation should be examined by the further studies.

Subjective memory complaints in the everyday memory function decreased more in the training group compared with the control group. The MMT provides users processes to utilize learned mnemonics during their daily lives and an opportunity to share their experiences with one another in each session. Therefore, memory complaints in everyday function, not the global memory function, would experience a greater effect from the MMT. In addition, subjective memory complaints are closely related with episodic verbal memory [23]. Since the MMT was effective at increasing verbal memory function, this may help relieve subjective memory complaints. 

This study had several limitations. First, we did not adapt the active control design. Further studies should include an active control group to control for placebo effects. Second, although the number of participants was larger than previous studies [10], a total of 113 older adults with MCI was not enough to find the interactions between the cognitive change and groups. In addition, the effects size of the MMT was small and the robustness of the finding was relatively weak. Therefore, the results of the current study provide preliminary evidence for the effect of MMT in older adults with MCI. Further studies with larger sample sizes are needed to increase the statistical power. Third, only the amnestic subtype of MCI was included in the current study, thus, we did not assess any effects in non-aMCI subtypes. A previous study demonstrated that the effects of training program (i.e., The Ubiquitous Spaced Retrieval-based Memory Advancement and Rehabilitation Training program) vary depending on the type of MCI (i.e., amnestic versus non-amnestic) in Korean older adults [24]; thus, further studies should be conducted to determine whether the effect of MMT varies depending on the MCI subtypes. Fourth, since the interval between the tests were 9 weeks, there could be a practice effect. Even though we compared enhancement of the training group with the control group, further research should examine the potential practice effects. Lastly, we did not carry out follow-up evaluation. Follow-up studies should be ongoing to further assess the longitudinal effects of MMT on MCI.

Despite these limitations, the performance data supported the short-term effectiveness of the MMT for improving cognitive functions (i.e., delayed free recall in verbal memory, categorical fluency) in older adults with MCI. The training also relieved complaint of everyday memory function. The MMT may help maintain the cognitive functions and mitigate inconveniences in older adults with MCI. 

## Figures and Tables

**Table 1:** Demographic characteristics of training and control groups.

	Groups				Total (*N* = 113)
	Training (*n* = 66)	Control (*n* = 47)	χ^2^ or *t*	*p*	
Gender (Male:Female)	39:27	20:27	3.01	0.090	59:54
Age (years)	70.83(5.02)	69.81(4.13)	1.15	0.253	70.41(4.68)
Education (years)	9.64(3.92)	9.74(3.52)	0.15	0.880	9.68(3.74)
Geriatric depression	5.18(3.95)	6.45(4.33)	1.61	0.110	5.71(4.14)
Mini mental state examination	26.33(2.53)	26.72(2.31)	0.84	0.404	26.50(2.44)

Note. The numbers in parenthesis are the standard deviation.

**Table 2:** Cognitive functions and subjective memory complaints across groups.

Cognitive Function	Training Group (*n* = 66)		Control Group (*n* = 47)		1: Main Effect of Cognitive Change 2: Interaction between Cognitive Change and Groups		Dependent *t*-test (pre vs. post) 1: Training Group 2: Control Group	
	pre	post	pre	post	*F* 1/2	*η*^2^ 1/2	*t* ½	Cohen’s *d* 1/2
**Verbal memory**								
Immediate free recall	3.88(1.99)	5.03(2.39)	3.87(1.90)	4.96(1.81)	27.02 ***/0.02 *^ns^*	0.196/<0.001	4.20 ***/3.25 **	0.52/0.47
Immediate cued recall	4.98(1.72)	6.27(1.95)	5.09(1.46)	5.91(1.27)	48.77 ***/2.28 *^ns^*	0.305/0.020	6.23 ***/3.92***	0.77/0.57
Delayed free recall	3.88(1.97)	5.29(2.54)	4.51(1.41)	5.06(1.70)	31.75 ***/6.04 *	0.222/0.052	5.58 ***/2.63 *	0.69/0.38
Delayed cued recall	4.79(1.93)	5.82(2.23)	5.21(1.44)	6.04(1.22)	32.89 ***/0.38*^ns^*	0.229/0.003	4.80 ***/3.48 *	0.59/0.51
**Visuospatial memory**								
Copy of Rey figure	15.14(0.96)	15.17(0.78)	14.98(1.05)	15.20(0.94)	1.73 *^ns^*/0.87 *^ns^*	0.015/0.008	0.29 *^ns^*/1.50 *^ns^*	0.04/0.22
Recall of Rey figure	10.25(3.83)	11.98(3.45)	11.10(2.30)	12.19(2.36)	32.20 ***/1.66 *^ns^*	0.230/0.015	5.30 ***/3.07 **	0.65/0.45
Recognition of Rey figure	16.58(2.14)	17.20(1.98)	16.87(1.80)	17.60(1.99)	9.35 **/0.05 *^ns^*	0.078/<0.001	2.09 */2.32 *	0.26/0.34
**Language**								
Naming test	11.33(2.34)	11.80(2.05)	10.94(2.27)	11.21(2.39)	12.68 ***/0.85 *^ns^*	0.103/0.008	3.52 ***/1.70 *^ns^*	0.43/0.25
Fluency	26.48(5.77)	28.09(6.02)	26.40(5.86)	26.57(5.52)	6.82*/4.11 *	0.054/0.036	3.47 ***/0.32 *^ns^*	0.43/0.05
**Attention**								
Digit score	7.98(2.00)	8.11(1.97)	7.94(1.84)	8.47(2.19)	5.35 */2.12 *^ns^*	0.046/0.019	0.68 *^ns^*/2.38 *	0.08/0.35
Digit back score	4.50(1.85)	4.45(1.99)	4.57(1.74)	4.72(1.64)	0.13 *^ns^*/0.46 *^ns^*	0.001/0.004	0.25 *^ns^*/0.65 *^ns^*	0.03/0.09
Visuospatial score	6.91(1.39)	6.61(1.45)	7.06(1.36)	6.85(1.59)	4.06 */0.12 *^ns^*	0.035/0.001	1.78 ^†^/1.14 *^ns^*	0.22/0.17
Visuospatial back score	5.18(1.89)	5.36(1.42)	5.36(1.42)	5.26(1.44)	0.02 *^ns^*/0.28 *^ns^*	<0.001/0.002	0.26 *^ns^*/0.55 *^ns^*	0.03/0.08
**Subjective memory complaint**								
Total	5.88(3.59)	4.94(3.23)	6.15(3.43)	5.98(3.57)	4.62 */2.22 *^ns^*	0.040/0.020	2.64 */0.48 *^ns^*	0.32/0.07
Global memory	2.76(1.04)	2.33(1.49)	2.67(1.28)	2.59(1.29)	4.10 */1.80 *^ns^*	0.059/0.027	1.83 ^†^/0.64 *^ns^*	0.40/0.09
Everyday memory	4.24(3.42)	2.52(2.44)	3.51(2.51)	3.43(2.68)	8.97 **/7.35 **	0.120/0.100	2.85 **/0.28 *^ns^*	0.62/0.04

Note. The numbers in parenthesis were standard deviation. *** *p* < 0.001, ** *p* < 0.01, * *p* < 0.05, ^†^ *p* < 10, *ns* = non-significant.
